# Premorbid risk perception, lifestyle, adherence and coping strategies of people with diabetes mellitus: A phenomenological study in the Brong Ahafo Region of Ghana

**DOI:** 10.1371/journal.pone.0198915

**Published:** 2018-06-14

**Authors:** Philip Teg-Nefaah Tabong, Vitalis Bawontuo, Doris Ningwiebe Dumah, Joseph Maaminu Kyilleh, Tolgou Yempabe

**Affiliations:** 1 Department of Social and Behavioural Sciences, School of Public Health, University of Ghana, Legon, Ghana; 2 Faculty of Public Health and Allied Sciences, Catholic University College of Ghana, Fiapre, Sunyani, Brong Ahafo Region, Ghana; 3 Catholic Education Unit, Sunyani, Brong Ahafo Region, Ghana; 4 Nurses Training College, Tamale, Northern region, Ghana; 5 Department of Surgery, Tamale Teaching Hospital, Tamale, Ghana; Florida International University Herbert Wertheim College of Medicine, UNITED STATES

## Abstract

**Background:**

One of the non-communicable diseases which is on the rise is type 2 diabetes (T2D). T2D is largely preventable with healthy lifestyle. We therefore conducted this study to explore premorbid perception of risk, behavioural practices and the coping strategies of patients with T2D.

**Methods:**

Using descriptive phenomenology approach to qualitative enquiry, we conducted eight focus group discussions (N = 73) with diabetic patients; four among males (N = 36) and four among females (N = 37). In addition, we conducted in-depth interviews with 15 patients, seven caretakers and three physicians. We adopted Colaizzi’s descriptive phenomenology approach to analyse the data with the aid of NVivo 11.

**Results:**

We found that respondents believed diabetes was a condition for the aged and rich and this served as a premorbid risk attenuator. Majority of them engaged in diabetes-related high risk behaviours such as lack of exercise, sedentary lifestyle and unhealthy eating despite their foreknowledge about the role of lifestyle in diabetes pathogenesis. We also found that patients used *moringa*, *noni*, *prekese*, and garlic concurrently with orthodox medications. Adherence to dietary changes and exercises was a challenge with females reporting better adherence than males. The study also revealed that patients believed biomedical health facilities paid little attention to psychosocial aspects of care despite its essential role in coping with the condition.

**Conclusion:**

Diabetic patients had low premorbid perception of risk and engaged in diabetes-related risky behaviours. Diabetic patients had challenges adhering to lifestyle changes and use both biomedical and local remedies in the management of the condition. Psychosocial support is necessary to enhance coping with the condition.

## Background

Diabetes mellitus is one of the chronic non-communicable diseases (NCDs) which is on the rise with high burden across the world. In 2013, 382 million were reported to have diabetes and this number is expected to rise to 592 million by 2035 [1]. In 2014, it was estimated that 422 million adults live with diabetes [2]. Although there are regional variations in diabetes, records available show that majority of the people with the condition live in low- and middle-income countries [3]. In 2010, the World Economic Forum ranked NCDs including diabetes among the most important threats to global economic development [4]. The prevalence of diabetes is reported to be higher in urban than rural areas [5,6]. Despite being a preventable condition, it has been estimated that, for persons aged 35–64 in Africa, diabetes claimed 7.1% of all deaths among males, and 7.9% among females [7]. In the year 2012, diabetes was responsible for 1.5 million deaths globally [2]. In Ghana, diabetes prevalence is 6.3% of adult population [8] and more than 450, 000 Ghanaians are living with the condition [9].

Although there are other types of diabetes, type 1 (T1D) and type 2 (T2D) diabetes have been reported as having the highest burden across the world. T1D which was formerly known as insulin-dependent diabetes cannot be prevented with current knowledge [2]. However, T2D also previously known as non-insulin dependent diabetes can be prevented through healthy behavioural practices. These lifestyle modifications are also important to prevent complications such as a kidney disease, heart attacks, musculoskeletal effects and premature deaths among people with the condition [10]. The risk of T2D result from an interplay between genetic and metabolic factors [11–13]. Nonetheless, some factors have been identified as increasing the risk of T2D. These include; ethnicity, family history of diabetes, overweight and obesity, unhealthy diet, inadequate consumption of dietary fibre, intake of sweetened beverages, lack of exercises, and smoking [14–18]. Although there are no separate global disaggregated data on the prevalence of these types of diabetes, T2D have been reported as the most common type of diabetes that affect people [2].

Perception of risk and lifestyle are important issues in diabetes prevention. Also, an individual’s ability to cope with the condition by adhering to medication, exercises and dietary requirements will reduce complications and deaths among people with T2D [19–23]. For example, it was found that adolescents with diabetes who understood the condition and made the necessary lifestyle adjustment were able to cope very well with the condition [24], a factor which is essential for blood sugar control. Poor adherence to treatment (both dietary and pharmacologic) affects the efficacy of the medications leading to inadequate glycemic control [25–28]. However, many of the studies on diabetes in Ghana from our review have often concentrated on the epidemiological aspect of the condition with little on the social science aspect. The aim of this study was to increase the understanding of the multifaceted psychosocial and cultural contexts of risk perception, premorbid behaviour, illness experience, management, adherence and social attitudes to diabetes.

## Methods and materials

### Ethics statement

Ethical approval was obtained from the ethical committee of the School of Medicine and Allied Health Science of the University for Development Studies, Tamale, Ghana. Approval was sought from the head of the health facilities to conduct the study. Written consent were obtained from all participants in this study. We also used pseudonyms to identify our participants during FGDs. The socio-demographic data were de-linked from the main data set to ensure anonymity.

### Study design

This study adopted descriptive phenomenology approach to qualitative research enquiry. Descriptive phenomenology is used to acquire an understanding of the true meaning of a phenomenon of interest through engaging in-depth with that reality [29,30]. This approach allows researchers to collect detailed data on a particular phenomenon of interest to gain deeper insight about the situation [31,32].

### Study area

The study was conducted in the Brong Ahafo Region (BAR) of Ghana. The region is bordered to the north by the Black Volta River and to the east by the Lake Volta, and to the south by the Ashanti region, Eastern and Western regions, and to the west by the Ivory Coast southeastern border. The region has a total population of 2,310, 983 [33]. The region is divided into 27 administrative districts [34]. BAR like all regions in Ghana runs a vertical public health care system from the community to the regional level. The smallest health care units at the community level are the community-based health planning and services (CHPS) zones or compounds managed by community health nurses. The next level of care is the community clinics/health centres with physician assistants (PA) or sometimes medical officer and midwives in-charge. The district hospitals are the third level of health care which are usually manned by medical officers. There are 22 district hospitals. Nine (9) of the district hospitals were established by the government and the remaining 13 were established by religious organizations. There is one Regional (referral) hospital located in Sunyani. This hospital is the main referral hospital for all the district hospitals, and runs a weekly diabetic clinic with Physician Specialists where diabetic patients from all over the region attend.

### Study participants and selection of participants

The study participants were patients with diabetes mellitus, their partners and/or caretakers and physicians who took care of diabetic patients. The researchers visited the regional hospital where there is ongoing specialized clinic for people with diabetes. Patients who attended the clinic over the study period were informed about the objectives of the study after they had received care from the specialist. The contact details of people who agreed to take part in the study were collected and an appointment was booked for the interview. All the interviews were conducted at the community level at times suitable for the respondents. However, patients located in different communities were brought together for group discussions at the nearest health facility. For respondents who had living partners, they were also interviewed. However, in some selected cases the persons who provide assistance to diabetic patients were rather interviewed (instead of the partner) alongside the patient. Specialists who took care of the patients were also purposively selected and interviewed.

### Data collection strategies

Focus group discussions (FGDs) and in-depth interviews (IDIs) were the main data collection strategy used in this study which was undertaken between October, 2016 and January, 2017. In all, eight FGDs (N = 73) were conducted among patients receiving treatment for diabetes stratified by sex and duration of treatment (Table 1). All interviews were conducted by the lead author (PT-NT) who at the time of the study was in the final year of his doctoral training in Public Health. He has a wealth of previous experience in conducting qualitative research and has consulted for both local and international organisations in qualitative study designs, data collection and analysis. During discussions, each individual had the opportunity to share his/her views on a question raised before moving to another question. This was done to ensure that all participated actively in the discussion. It took between 60–90 minutes to complete a discussion. Detailed field notes were written on demeanor of respondents, facial expressions and the environment the study was conducted.

**Table 1:** Summary of FGD participants.

Categories	Males		Females	
	Number of FGDs	Number of Participants	Number of FGDs	Number of Participants
≤5 years	2	18	2	18
>5 years	2	18	2	19
Total	4	36	4	37

Face-to-face IDIs were the second method of data collection and are believed to be capable of providing more private information [32,35]. Twenty-five (25) IDIs were conducted whereof 15 were diabetic patients, seven partners/caretakers, and three Physician Specialists. It took between 45–60 minutes to complete an IDI. The researchers first conducted 13 IDIs with diabetic patients and the data was analysed. At that point we realized that there were no further new ideas emerging. However, we decided to add an additional two interviews to compare with the 13 interviews. The comparison with the initial 13 interviews and FGDs revealed that we had reached the point of saturation [32] since no new insights emerged in the two IDIs.

Participants were not biologically or socially related to any of the researchers. Two of the researchers (PT-NT and VB) who are males have previously worked in some health facilities in Ghana but at the time of study were not directly engaged in clinical care of patients. PT-NT is a trained nurse whilst VB is a biomedical scientist who now work as public health researchers. DND is a female with background in food science and a teachers. JMK is nurse educator working in a different region and currently not involved directly in clinical care of patients. TY is an orthopaedic surgeon with interest in public health research in his specialty area.

The study was conceived following discussions by the researchers. PT-NT, DN and VB had an initial discussion about the increasing number of diabetes cases in Ghana which they believed could be due to unhealthy lifestyle of individuals. In a subsequent conversation between PT-NT, JMK and TY, it emerged that there were increasing number of diabetic foot cases reporting at the outpatient department of the hospital TY works. PT-NT who is now a social scientist then proposed a study in the social science aspect of the condition.

### Data collection tools

Semi-structured FGD and IDI guides were used for data collection. The tools for both FGDs and IDIs with patients were initially designed in English and later translated to Twi by a language expert. The tools were pretested in a pilot study in the Greater Accra region of Ghana. Five diabetic patients, five caretakers and two physician specialists were recruited for the pilot study. These guides covered areas such as family history of diabetes, premorbid perception of risk, lifestyle, health seeking behaviour, initial reaction to diagnosis, intra-family and community experience and coping strategies. It also elicited information on health system experience such as client-provider interaction, provision of health education, quality of health care and adherence. The IDI guide for Physician mainly focused on health system related factors. However, after interviewing some patients and caretakers, emerging issues were incorporated into the tool during data collection for Physicians.

### Data analysis

**Fig 1:**
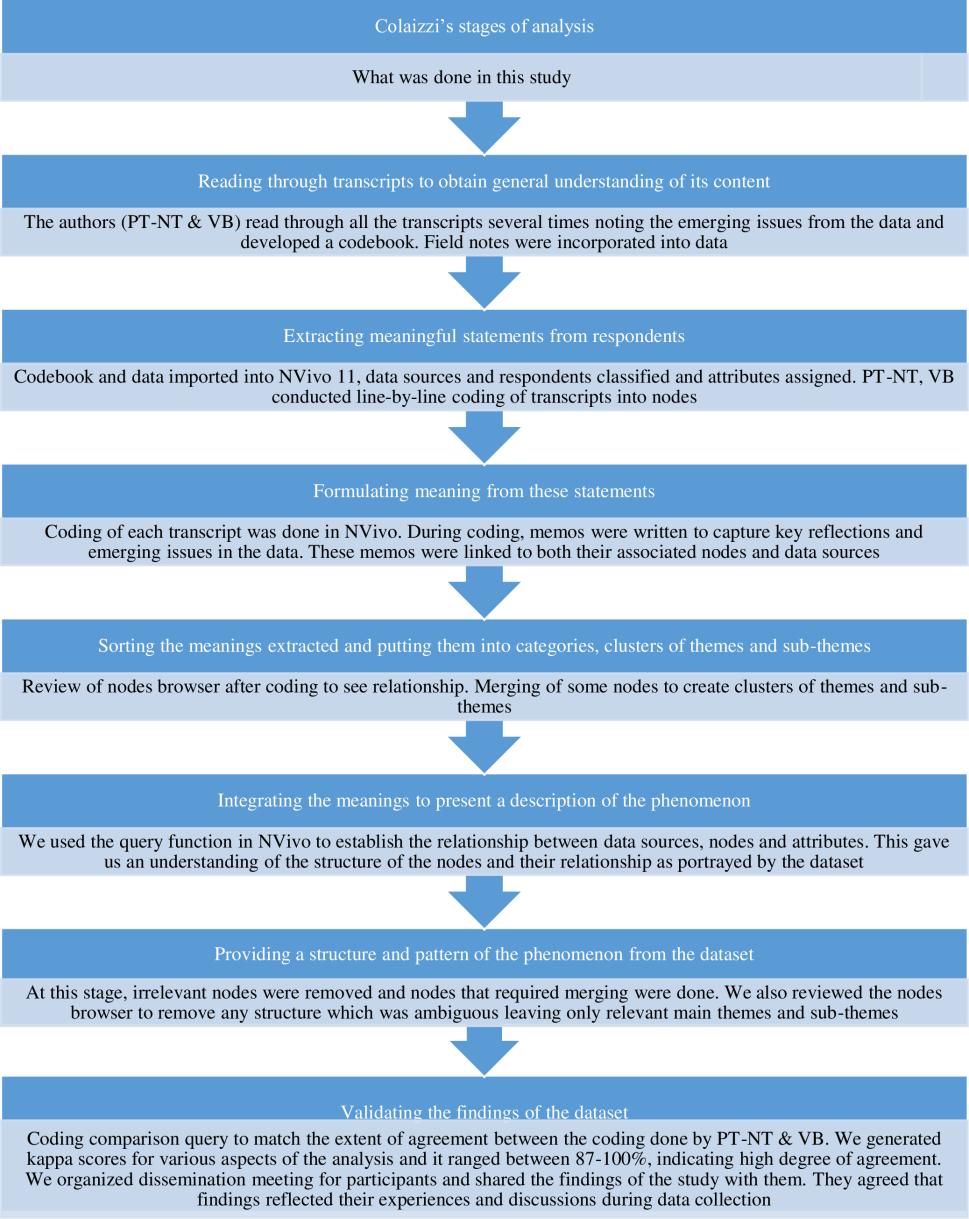
Colaizzi’s stages in descriptive phenomenological analysis and how it was applied in this study.

We adopted Colaizzi’s descriptive phenomenology data analysis strategy. There are seven processes/steps involved in analysing qualitative data using this approach [36], (Fig 1). Bracketing was used to ensure that the interpretation represented the experience of respondents [37]. The results are presented in narratives and supported with the most telling illustrative quotes from respondents as required in phenomenology [31].

## Results

### Background characteristics

In all 88 patients with diabetes participated in this study. Majority (51%) of them were males, and were married (62%). About 59% had family history of the condition and 52% have been on treatment for this condition for more than 5 year (Table 2).

**Table 2:** Socio-demographic characteristic of diabetic patients.

Socio-demographic characteristic	Frequency	Percentage
**Diabetic Patients (N = 88)**		
**Sex**		
Male	45	51.0
Female	43	49.0
**Age**		
<40 years	31	35.0
≥40 years	57	65.0
**Marital status**		
Married	55	62.0
Single	21	24.0
Divorced	7	8.0
Widowed	5	6.0
**Religion**		
Christianity	55	63.0
Islam	33	27.0
**Family history of diabetes**		
Yes	52	59.0
No	16	18.0
No idea	20	23.0
**Duration of condition**		
≤5 years	36	41.0
>5 year	52	59.0

### Premorbid perception of risk and lifestyle

The study found that majority of the respondents perceived that they were not vulnerable to diabetes. This belief was held by 56 out of 73 (76%) FGDs participants, and 9 out of 15 (60%) diabetes patients who were interviewed individually. Even among those with family history of diabetes, the general perception was that they were not vulnerable to the condition. For those who perceived higher risk of diabetes, the perception was related to close relatives and people they generally shared lifestyles with getting the condition. For instance, one FGD participant reported:

*“I never believed I could get the condition diabetes*. *In fact people have been talking about the condition and how common it has become*. *However*, *if someone had told me that I could get the condition*, *I would never have believed that person*. *So when the doctor first told me about the condition*, *I could not believe it and refused to take the medicine until things were getting worse”* (46-years female, FGD).

In a separate FGD, another participant reported:

*“Some of my family members were diabetics but I did not believe that I could get it until my close friend that I share everything together with got the condition*. *We used to eat and drink beer together*. *So when he told me the doctor said he had diabetes and knowing very well that it is a condition related to the food we eat*, *I became afraid until finally I was also diagnosed of having the condition*” (67 years male, FGD).

The results also showed that many, 82 of 88 (93%) of diabetic patients indicated they were not doing any exercise prior to getting the condition. Generally, the study showed that all the respondents had received health education and information on the need to do regular exercises but did not deem it so necessary. One interview participant shared his experience in the following quote:

*“As for the exercise*, *I must be frank I have received information on it but it was difficult to practice*. *Because we have diabetes in our family*, *my father died from the condition*, *so my wife used to advise me to do exercises and to stop taking alcohol but I did not take the advice serious until I got the condition*. *I am sure it could have made a difference but is too late”* (68 years male, IDI).

We also found that majority of the respondents indicated they used to take both alcoholic and sweetened drinks despite foreknowledge about this lifestyle increasing vulnerability to diabetes. To respondents, knowledge about these predisposing factors does not necessary make it possible for people to avoid such. The following quotes illustrate these points:

*“…*.*Before I got the condition*, *I was well aware that taking sweetened beverages could increase ones vulnerability but abstaining from it was a challenge*. *As a Muslim*, *I do not take alcohol so it is the sugar drinks that we can take”* (72 years, male, FGD).

*“I used to take a lot of fast food*. *The nature of my work makes it difficult for me to cook*, *so I rely on food sold outside*. *But now I am careful with what I eat; less fat*, *meat and less alcohol”* (36-years, female, IDI).

The study further found that diabetes was believed to be a condition for the aged and rich in the community. This believe resulted in a reduction in respondents risk perception about the condition. Two participants in separate FGDs shared their beliefs about diabetes:

*“Before I had the condition*, *I used to think that the condition was for rich and old people*. *So this made me not to take exercise*, *and unhealthy diet serious”* (38 years female, FGD).

*“You see in my community*, *we believe that diabetes is a condition for rich people*. *So when I got the condition some of friend asked how did I get this rich men condition*. *So we don’t take the health education serious because of the perception that we cannot get the condition”* (58 years male, FGD).

The study also explored participants’ awareness of premorbid diabetes risk assessment tools such as Finnish Diabetes Risk Score (FINRISK) Tool, Australian Type 2 Diabetes Risk Assessment Tool (AUSDRISK) and Indian Diabetes Risk Score (IDRS). It emerged that no respondent was aware of these tools. For instance, one participant confirmed in a FGD as follows:

*“I have never heard of this tool*. *So you mean using that tool could help assess our risk and therefore adopt better lifestyle so that we don’t get it*? *It would have been helpful*. *I think health workers should educate people on this tools”* (52 year male, FGD).

Another participant indicated in a separate FGD:

*“You see*, *the Bible says for lack of knowledge my people perished*. *This tool could help*. *Even though I never perceived myself vulnerable*, *If I were to be aware of the tool*, *I will do it and if I see that I will be vulnerable*, *I will be careful about what I eat and drink”* (43 years female, FGD).

### Health seeking behaviour prior to diagnosis

The findings of this study showed multiple health outlets were used by people with diabetes prior to formal diagnosis of the condition. These include biomedical, traditional medical practitioners, spiritualists and home-based remedies. Many of the respondents (80%) indicated that these were often sought simultaneously or in sequences based on the pieces of advice they received from people. The sequences according to respondents was either biomedical to traditional or traditional to biomedical. Others also combined biomedical with spiritual or traditional with spiritual. However, female respondents used spiritual help outlets than their male counterparts. After the diagnosis, many indicated they use the biomedical remedies alongside some herbs and home remedies such as *prekese* and garlic. This is illustrated by:

*“You know when you have a problem*, *you get advice from several people*. *So in that distressing situation you obey whatever advise you get*. *I initially bought some herbs from itinerant herbal doctor which many believed could control blood sugar*. *I took it for some time before going to the hospital*. *Now I take both herbs and insulin*. *The herbs are natural and do not conflict with insulin”* (69 years, male, FGD).

*“After the diagnosis and the treatment by the doctor*, *I was advised to see pastor [named removed] because he has been healing people on television*. *You know some of these conditions can be spiritual*. *So I went there for some anointing oil and holy water*. *So I am combining the two and is helpful*. *The pastor even asked me to stop the medicine from the doctor but I think combining the two is better”* (63 years female, IDI).

From the perspective of caretakers, one interview participant also indicated:

*“When my father’s condition started we went to see a pastor*, *hospital*, *and herbal treatment*. *Where did we not go*? *But now he is getting better on the medicines from the hospital*. *We also use home-based remedies like prekese*, *noni and garlic”* (28 years male caretaker, IDI).

### Reaction to the diagnosis of diabetes

The study found that many initially felt uncomfortable when they were told they had diabetes because it is a chronic condition which requires a lifelong management and daily medication. The most troubling part was the dietary requirements and lifestyle changes associated with the disease condition. This, according to respondents in this study, saddened their hearts and left them thinking for several days. However, this feeling gradually faded when they commenced the treatment. An FGD participant reported:

*“…*.*You know it is not easy to be on medication for your entire life*. *So initially I kept thinking about the condition and this made me to grow lean*. *As an adult*, *you are told to only eat some foods and just some number of pieces*. *It is really worrying”* (46 years female, FGD).

Some respondents especially those below the ages of 40 years stated they denied the condition initially because of the belief that diabetes was a condition for the aged as illustrated:

*“I initially told myself that it was impossible because I was young about 35 year*. *My thinking was that this disease is for the aged and rich in society*. *So how can a poor man like me get*. *It actually took me over a year to come into terms with the fact that I was diabetic”* (42 years male, FGD).

Physicians interviewed in this study indicated the need for counselling and psychological support for patients to accept their condition. They recommended that psychologists be posted to hospitals, so people with such chronic conditions that require lifelong therapy could receive some psychotherapy and support. For instance, one Physician specialist reported this in an interview:

*“We as doctors educate them about the condition and the fact it requires a lifelong daily medications*. *We also refer them to dieticians for information on diet*. *Many of these clients will definitely have some psychological problems*. *Just the thought of taking medications for their entire life is stressful”* (Physician 1, IDI).

Another Physician specialist interviewed shared his opinion on psychotherapy for diabetic patients as follows:

*“If we have psychologist in our facilities*, *we could refer some of the cases to them*. *If they are available all new cases could be referred to them for some psychological support and management”* (Physician 2, IDI).

### Home care/management of diabetes

The study found that respondents were generally happy with home management of the condition. All the respondents reported they regularly take their medications with the help of their partner or family caretaker. Additionally, some diabetic patients had their personal glucometer which they use to check their blood glucose level at home. This is illustrated by:

*“It is not easy to live with this condition but I am able to take my medications regularly*. *I have the machine for checking the sugar*, *so I check it regularly”* (46 years male, FGD).

*“I was trained at the hospital on how to give the medicine to my husband*, *so I usually give it to him daily*. *Initially we did not have the machine to check the sugar but with the grace of God we have been able to buy one and it helping us a lot”* (45 years female, IDI).

Respondents indicated the importance of having a personal glucometer to monitor glucose level at home despite financial challenges in procuring one as illustrated:

*“My daughter is a nurse who still lives with me*, *so she is able to give the injections at home and also check my sugar level for me*. *It is very helpful if everybody can get the machine*. *This condition has become common so the government should subsidy the machine and strips to make it affordable for everybody with the condition to buy”* (68 years male, FGD).

*“Getting money to buy insulin and the test strips is a major problem*. *I have the machine but as we are talking today I have not been able to check my blood glucose level because I have no money to buy the strips”* (63 years female, FGD).

Additionally, the findings of this study revealed that diabetic patients introduced local remedies (alongside taking their medications) as part of home-based management of the disease condition. About 60% of diabetics in this study combined both orthodox and local remedies to manage the condition at home. Some of the local remedies mentioned in this study included *moringa*, *prekese*, *noni (Morinda citrifolia)* and garlic. *Prekese* is a Twi (language) word for *Tetrapleura tetraptera* plant whilst *moringa* is derived from *Moringa oleifera* plant. Other participants also reported that they combined orthodox medicines with some concoctions given to them by traditional healers. These local remedies are believed to be capable of reducing blood sugar level. The following quotes illuminate these points:

*“Apart the injections I take daily*, *I also take prekese*, *noni and moringa which is good for our health*. *Prekese is able to reduce blood sugar*. *A friend who is also having the condition recommended these things to me”* (48 years male, FGD).

*“Many of us combine the Whiteman medicine with some local medicine and herbs*. *Some of us also use garlic*, *and prekese*. *These things can help prevent both diabetes and hypertension”* (38 years, female, FGD).

*“I combine the medicines from the hospital with some herbal medicine from one herbalist*. *I was actually taking that medicine before the diagnosis at the hospital*. *When I started experiencing the symptoms*, *I went to the herbalist who told me I have sugar disease and gave me the concoction*. *I came to the hospital because I had a sore which could not heal”* (68 years male, IDI).

### Adherence to lifestyle changes and coping strategies

It was widely reported that adherence to lifestyle changes as part of managing diabetes was essential; however the findings in this regard varied. Whereas some participants generally reported their ability to conform to lifestyle changes in the area of physical exercises and diet, others were uncomfortable and could therefore not cope with the changes. Such individuals reported challenges in adhering to the dietary and other behavioural changes required to control blood sugar. One participant reported:

*“…it is very difficult to change your diet and only eat what has been prescribed but what do you do*. *You either comply or suffer the consequence*, *so for me I obey and stick to what I have been told to eat”* (47-years female, IDI).

Another participant revealed family constraints associated with lifestyle changes and adherence as a diabetic patient:

*“I am able to cope with the dietary prescriptions from the dietician*. *Initially*, *it was a bit challenge because it has affected the family pot and my wife had to adjust menu in the house”* (68 years, male, FGD).

The study found that females were adhering better to changes in lifestyle and behaviour compared to their male counterparts. Out of the 45 male patients that participated in this study, only 25 (55%) of the patients indicated they were able to adhere to dietary requirement, exercises and self-blood sugar checks in the week prior to the study. However, 32 out of the 43 females (74%) were able to adhere to these three important elements in diabetic-related care. In FGDs, males generally expressed challenges in adhering to these lifestyle changes relative to females. One male participant shared his experience with lifestyle changes:

*“For us males*, *it is difficult to adhere to the lifestyle changes*. *The females are doing better*. *My wife also has the condition and is able to follow what the doctors and the dieticians have told us*. *She is the one encouraging me to cope but it is difficult”* (48 year male, FGD).

The study participants also reported the benefits of social support system as part of coping mechanisms in the life of a diabetic patient. Many participants (87%) coped with social support systems such as networking with friends, relatives, religious bodies, and social clubs; and also relying on their religious values. It was reported that these networks encouraged them to take their medications and also provide them with some financial support. These networks also created opportunities for them to engage in diverse activities which took away their worries about the condition. This is illustrated by:

*“I belong to several social groups both in my community and in church*. *You engage yourself to forget about the worries of your condition*. *Today is weekend*, *I just returned from one of such meetings”* (53 year, female, FGD).

In addition, it emerged from the study that the Ghana Diabetes Association, an association for people with diabetes, provided good social support to people with diabetes through regular meeting and discussions about the condition as illustrated:

*“We get support from the diabetes association*. *It is good to meet people who have the same condition as you to share experiences and the way forward*. *This makes you feel that your experiences are not unique*. *So if other people also have similar experiences and go on with their lives why worry*? (63 year male, FGD).

In addition, social groups provided support to diabetics with health walks and playing of long tennis as physical exercises. This following quotes support this:

*“I belong to social group and we organize health walks during the weekend and this help me to do some exercise*. *I believed if I had joined this group earlier*, *I would not have gotten this condition”* (45 years male, FGD).

*“We go to play table tennis and long tennis with our peers during the weekend and this serves as diversional therapy and exercise*. *I recommend everybody to join such clubs*. *They will encourage you to do some exercises”* (48 years male, IDI).

We also observed some challenges at the family level concerning libido loss and ability to satisfy their partner for both men and women with diabetes as illustrated:

*“You know I have both hypertension and diabetes*, *so the condition and the medications make me unable to sustain an erection for long*. *So*, *I am not able to satisfy my wife these days*. *I am really concern about this because this can make your wife go behind you to get the satisfaction that you are unable to provide”* (39 years male, IDI).

*“My main concern is having sex with wife*. *It is gradually becoming difficult to go the number of runs I use to go*. *You set a standard and your wife has become used to the standard but you cannot keep it*, *what happy (laughing)*? (41 years, male, FGD).

*“I support my husband very well with his condition but the only problem is that he does not have the feeling for sex any longer and is not able to perform this days but we have pledged to remain faithful to each other”* (34 year female partner, IDI).

### Health system experience of diabetic patients

The study reported varied health system experiences among patients with diabetes. Majority of the respondents generally had a positive health system experience. Many reported they were treated very well at the health facilities. Participants also reported that they have regular supply of medications. A FGD participant shared his experiences with the health system emphasising on respect for privacy as follows:

*“We receive very good care from the hospital*. *They will receive you well and respect your privacy*. *So for me I am generally happy with the care*. *I will give them 90% if I were to mark them”* (68 year male, FGD).

A participant in another FGD indicated that she was impressed with regular supply of drugs from the hospital as follows:

*“I am happy with care at the hospital*. *I receive my drug regularly except the insulin that I buy from the pharmacy shop*. *Both the nurses and doctors treat us well”* (45 years, female, FGD).

Nevertheless, it was generally reported that there are delays in receiving care at the health facilities. This is because patients come early to the clinic to test their sugar at the laboratory but have to wait for a long time before receiving treatment. A participant indicated in the following quote:

*“One area that the hospital need to improve is the waiting time*. *We come here in the morning to queue for long*. *If the clinic can start early*, *it will help us”* (62 years male, FGD).

Additionally, participants expressed concerns about the lack of psychological care at health facilities for diabetic patients. This, in their view, was necessary to facilitate adherence to treatment, coping with the condition and lifestyle changes as illustrated:

*“We do not receive psychological support at the hospital*. *Some counselling is required to enable us cope with the condition*. *This could be extended to our partner*. *Just as my brother said earlier*, *because of the condition you may have challenges performing your marital duties at home*.” (46 years male, FGD).

We found that patients were informed about regular checking of their blood sugar, injection of insulin and adherence to treatment as illustrated:

*“When I was diagnosed with the condition*, *the doctor explained to me the condition and stressed on the need for me to regularly check my blood sugar and take the medication*. *I was advised to buy the machine so that they will teach me how to use which I did”* (64 years male, FGD).

“*The nurse in the consulting room of the doctor asked me if I had money to buy the machine so that I could check my sugar at home since I lived far away from the hospital*. *I bought it and came to the hospital with my son and they educated him on how to use the machine*. *So it is good”* (63 years female, IDI).

## Discussion

We conducted a qualitative study with a descriptive phenomenology approach. We found that diabetes is perceived as a condition for the aged and rich in community. The findings also generally showed that diabetic patients had a low premorbid perception of risk, and hence engaged in diabetes-related high risk behaviour. Prior to diagnosis, the participants also used both orthodox and non-orthodox health facilities. Patients also used local remedies or engage in self-medication in addition to treatment they receive from biomedical facilities. Our study further showed that patients had challenges adhering to dietary requirements, exercises and home-based blood sugar monitoring. The study underscore the need to find innovative ways to encourage patients to adhere to treatment. Education and community sensitization on diabetes should highlight the fact that all individuals irrespectively of their social class and age are vulnerable to diabetes.

### Premorbid risk perception and lifestyle

The study showed that respondents did not perceive risk of diabetes prior to the diagnosis. This was also the case for people who had a family history of the condition. Family history of diabetes increases an individual’s vulnerability to the condition. In their study, it was found that a family history of T2D was associated with a higher incidence of the condition with people with bi-parental history of T2D having the greatest risk [38]. Another study found that 27% of people with T2D reported at least one relative with diabetes with more prevalence among people whose direct parents had diabetes than other relatives [39].

The perception that diabetes was a condition for the aged and rich emerged as risk attenuator in this study according to Kasperson’s Social Amplification and Attenuation of Risk framework [40]. This belief made people to engage in diabetes-related high risk behavior such as sedentary lifestyle and unhealthy eating. Yet, diabetes can affect people of various socio-economic status and a study in Ghana found that T2D tend to affect mostly obese patients of relatively low socio-economic status [41]. Health education on diabetes should highlight the risk of the condition across people of all ages and socio-economic status.

Exercises, healthy diet and activities to reduce weight can reduce the risk of T2D to 50–58% among high risk populations [42,43]. Ghana developed a regenerative health and nutrition programme to encourage healthy lifestyle and eating among Ghanaians by providing health education on good eating habits and exercises [44]. However, the findings of this study indicate an evaluation of this programme would be necessary. Just providing information about this without measures or creating a platform for people to adopt healthy lifestyles may be ineffective as all patients in this study had received education on healthy lifestyles. To be able to achieve the global target of halting the rise of diabetes and obesity [45], new strategies will be necessary. For example, workplace interventions that target healthy dietary and physical exercise are effective [46] and availability of healthy diets at restaurants and eatery can promote healthy food practices [4]. Some of these strategies could be adopted in addition to enforcing the Public Health Acts [47] to reduce the effects of smoking and alcohol.

The study generally found that respondents were unaware of the existence of tools that could be used to assess an individual’s risk of getting diabetes. A number of tools exist that can help one assess his/her risk. These include: FINRISK [48], AUSDRISK [49] and IDRS [50]. These tools can provide relevant information of an individual’s premorbid risk of diabetes and could lead to the adoption of healthy lifestyle. It is therefore important for health education on diabetes to highlight the availability of these tools. The respondents in this study clearly indicated they could have adopted better healthy practices if the use of the tool showed they were vulnerable to developing diabetes in future. The use of these tools could also improve health seeking and help in early diagnosis if properly used in addition to other diagnostic tests.

### Reaction to diabetes diagnosis and home management

We found that patients do not receive enough psychological support despite the fact that many reported some negative feelings towards the condition. Psychological care therefore requires attention and diabetic patients should be referred to psychologists for counselling and psychological support. Studies have shown high rates of anxiety and depression among patients with diabetes [51–54]. The findings of this study underscore the need to attach psychologists and counsellors to diabetic clinics to provide patients with the psychosocial support they require. For chronic conditions such as diabetes, it would be important to move beyond the clinical aspects to include other aspects of care such as psychosocial that are essential to facilitate coping. This study found some psychological distress among diabetic patients in the area of loss of libido which may have the potential to cause intra-spousal conflicts. Hence, psychosocial care should be integrated into the clinical for diabetic patients.

Diabetic patients also use both orthodox and non-orthodox treatment in the management of the condition. Patients use *prekese*, *noni*, *moringa* and garlic as these are believed to be capable of controlling blood sugar. Others studies have also reported on the use of herbal plants in diabetes control in China [55], Guinea [56], Iran [57]and Pakistan [58]. Although the efficacy of these plants (prekese, moringa and garlic) in controlling blood sugar are not known except for noni [59], it would be important for studies to be conducted on these plants as they are widely used in Ghana in combination with orthodox medicines patients receive from biomedical health facilities. Further research is required to ascertain their composition to inform health education on diabetes prevention and control.

### Adherence to lifestyle changes and coping strategies

Our study found that patients had challenges adhering to dietary requirements, exercises and home-based blood sugar monitoring. In addition, the study found that females were adhering better to changes in lifestyle and behaviour compared to their male counterparts. An earlier study among diabetic patients in northern Ghana found that adherence to dietary requirement and self-blood sugar monitoring was low and level of education was a key determinant to adherence [60]. Despite the contextual difference between Ghanaians living in northern and southern Ghana, common challenges in adherence to blood sugar control measures exist. Our study has however found gender differences in adherence to these lifestyle changes. The findings of this study underscore the need to pay more attention to male patients with diabetes to encourage them to adhere to the lifestyle changes.

Our study also found that a number challenges and ways employed to circumvent these challenges as coping strategies. First and foremost, the findings of the study revealed that people with diabetes have financial challenges. Financial challenges affect patients’ ability to adhere to follow-up reviews, purchase of glucometer strips to regularly monitor blood sugar levels, and buying of insulin. Some had to rely on other relatives for financial assistance. In an earlier study, it was reported that receiving financial support from immediate and distant family members was common among people with diabetes [61]. Another study also found that people with T1D expressed similar financial challenges [62]. It would therefore be important for the government to consider subsidizing glucometer and strips for diabetics as suggested by respondents in this study.

Secondly, it was reported that many rely on religious values and social support systems to be able to cope with the condition because it requires lifelong treatment. Social support is essential in the management of chronic conditions. These social networks also provided platform for people with diabetes to share their experience and an avenue for physical activity. As has been observed, the advent of non-communicable disease has created a situation where people have to spend substantial parts of their lives in what is described as “less than perfect health” because they have to cope with a chronic condition [63]. This findings also underscore the need for continuous psychosocial support in the management of diabetes.

## Study limitations

Although this study provides evidence on illness perception, lifestyle, health seeking and adherence to treatment that is required to guide policies on health promotion and interventions, it is important to situate the conclusions in the context of some limitations. The first limitation of this study is that it was conducted in one region in Ghana on a sample that is not representative, hence the findings cannot be generalized. This notwithstanding, in conducting the study, we followed the methodological requirements for a qualitative research such as the RATS checklist [64], Consolidated criteria for reporting qualitative research (COREQ), [65] and acceptable practice in fieldwork, analysis and interpretation [66].

The second limitation is that some of the IDIs and FGDs were conducted in the local language and translated during transcription. It is therefore possible that some words and phrases may have been lost their meaning as a result of the translation. To circumvent this weakness, two people were made to do the transcription for each interview and compared. Where there were disagreement, the authors discussed the translations with the two transcribers to arrive at the correct representation. All authors had foreknowledge of the local language. During analysis, emphasise was also placed on emerging themes and not the individual word choices or phrases. In select cases the local words used by the participants were retained during transcription.

## Conclusions

Diabetic patients in this study had low premorbid perception of risk and engaged in diabetes-related risk behaviour prior to the diagnosis. Associating diabetes with some socio-economic class served as a risk attenuator and leads to denial of the condition. Both biomedical and local remedies are used concurrently in the management of diabetes among study participants. Furthermore, diabetic patients have challenges living with the condition and adhering to lifestyle changes required to control blood sugar. However, social support was necessary to enhance coping with the condition.

## Abbreviations

AUSDRISK: Australian Type 2 Diabetes Risk Assessment Tool
BAR: Brong Ahafo Region
CHPS: Community-based Health Planning and Services
FGD: Focus group discussion
FINRISK: Finnish Diabetes Risk Score
IDI: in-depth interview
IDRS: Indian Diabetes Risk Score
NCDs: Non-communicable Diseases
PA: Physician Assistant
T1D: type 1 diabetes
T2D: type 2 diabetes
